# Medical News and Bulletin

**Published:** 1843-06-10

**Authors:** 


#### ST. LOUIS MEDICAL AND SURGICAL JOURNAL.

We have received the first number of this journal, edited by Professor M. L. Linton. It is the first medical journal established west of the Mississippi. It is to be published monthly. Its form and appearance are very neat, and its contents varied and interesting. Each number will contain the monthly meteorological observations of Dr. B. B. Brown, which will be useful and valuable.

---

The Western Lancet, edited by Dr. Lawson, and published at Cincinnati, has commenced its second volume. It is a well conducted, spirited monthly, and will, we hope, obtain the success it merits. The last number contains an excellent article on the importance and necessity of private insane retreats, from the pen of Prof. Mitchell, of Transylvania University; a history of the Dengue, as it occurred in Greene county, Ohio, in the spring and summer of 1842, by J. Dawson, M. D.; with a sketch of the late Dr. Oliver, by Professor Mussey.

---

#### BOOKS RECEIVED.

We have received the following publications, notices of several of which, prepared for the present number, we were obliged to omit from want of space. 1. Transactions of the College of Physicians. 2. Berzelius on the Kidneys. 3. Harris on Diseases of the Maxillary Sinus. 4. Andral's Medical Clinic, by Spillan. 5. Evanson and Maunsell on the Diseases of Chiidren, by Condie. 6. A Treatise on Consumption, by W. A. McDowell, M. D.

---

#### ELECTION OF M. VELPEAU TO THE FRENCH INSTITUTE.

M. Velpeau has been elected to supply the seat vacant in the French Academy of Sciences by the death of Baron Larrey. The chief candidates were MM. Velpeau, Lallemand, Lisfranc, and Civiale. On the first ballot M. Velpeau received 20 votes; M. Civiale 15; M. Lallemand 14; M. Lisfranc 6. On the second ballot, M. Velpeau had 26 votes, and M. Lallemand 22. Finally, on the third ballot, M. Velpeau obtaining 33 votes, and M. Lallemand 26, the former gentleman was declared duly elected. It is worthy of remark that M. Civiale obtained 15 votes, although his name had not been presented in the list of candidates by the Committee of the Academy.

---

#### THE PHYSICIANS OF DANTZIC SIXTY YEARS SINCE.

The character of our Dantzig physicians of that day left my father not the faintest hope of effecting his purpose by their means. In the first place, they were all and several extremely old, and petrified in obstinate prejudices. Whether they had ever been young, where they had lived, and what they had done in their youth, I know not; but I can affirm, that up to the twelfth or fourteenth year of my life, I had never seen nor heard of a young physician. These reverend gentlemen enjoyed the title of excellency, and not only in their own houses and from their own servants, but in society generally; only very intimate friends could sometimes venture on a respectful "Herr Doctor." Their head was covered by a snow-white, powdered, full-bottomed periwig, with three tails, one of which hung down the back, while the others floated on the shoulders. A scarlet coat embroidered with gold, very broad lace ruffles and frill, white or black silk stockings, knee and shoe buckles of sparkling stones, or silver gilt, and a little, flat, three-cocked hat under the arm, completed the toilette of these excellencies. Add to this a pretty large cane, with a gold head, or mermaid carved in ivory, upon which, in difficult cases, to rest the chin—and certainly every one will admit the impossibility of so much as thinking of an innovation in their presence.—*Edinburgh Review*, Feb. 1843, from Mad. Schopenhauer's *Jugendleben.*

[The innovation alluded to is inoculation of the small-pox.]

---

#### SUPERIOR ARRANGEMENTS IN GERMAN HOSPITALS.

It is impossible not to be struck with the superior degree of attention that the German surgeons bestow upon every minute circumstance which bears upon the success of their operations. This is well seen in the ophthalmic wards of their general hospitals. For example: the auditorium for the clinical practice in the great civil hospital in Vienna is coloured green, and the windows are supplied with peculiar shutters and curtains, so that the light can in a moment be regulated according as circumstances demand. The eye wards are likewise coloured green, and are furnished with everything necessary for the peculiar care of patients with eye-diseases: they have their special shutters and curtains, and each bed has a moveable screen to regulate the light admitted. Similar remarks apply to all the German hospitals, as those of Munich, Prague, Leipsic.—*Dr. Hamilton in Monthly Journal of Medical Science.*

---

#### SPERMATOZOA OBSERVED A SECOND TIME WITHIN THE OVUM.

Dr. Martin Barry has addressed the following note to the Editor of the London Medical Gazette.

"Several months since I communicated to the Royal Society the fact that I had observed, and shown to Professor Owen and others, spermatozoa *within* the mammiferous ovum. The ova were those of the rabbit, taken twenty-four hours *post coïtum* from the Fallopian tube.

"I have this day confirmed the observation; several ova from the Fallopian tube of another of these animals, in a somewhat earlier stage, having presented spermatozoa in their interior, *i. e.* (as in the first observation) within the thick transparent membrane ('*zona pellucida*') brought with the ovum from the ovary."

---

#### DEATH OF DR. BULLARD.

Died, at Dresden, in the thirty-eighth year of his age, Dr. Bullard, a gentleman well known by his labors relative to the plague, to the study of which he had devoted the greater part of his life in Turkey and in Egypt.

